# Tuberculosis masquerading as malignancy: a multimodality approach to the correct diagnosis – a case report

**DOI:** 10.1186/1477-7800-2-10

**Published:** 2005-05-07

**Authors:** Shalini Amukotuwa, Peter FM Choong, Peter J Smith, Gerard J Powell, John Slavin, Stephen M Schlicht

**Affiliations:** 1 Department of Medical Imaging, St. Vincent's Hospital, Fitzroy 3065, Melbourne, Australia; 2 Department of Orthopaedics, St. Vincent's Hospital, Fitzroy 3065, Melbourne, Australia; 3 Department of Pathology, St. Vincent's Hospital, Fitzroy 3065, Melbourne, Australia

## Abstract

**Background:**

Extrapulmonary tuberculosis is one of the great mimickers of medicine, and often masquerades as malignancy. As a result, patients may be referred to oncologists and surgeons for further evaluation and management, delaying the institution of appropriate anti-tuberculous drug therapy.

**Case presentation:**

We present the case of a 21 year old man with tuberculous osteomyelitis, who was referred to the Bone and Soft Tissue Sarcoma Service at our institution with a provisional diagnosis of malignancy. Further investigation revealed extensive retroperitoneal abdominal and pelvic lymphadenopathy. The recognition of certain patterns on imaging, and finally the isolation of *Mycobacterium tuberculosis *from tissue samples obtained under image guidance, enabled the correct diagnosis to be made.

**Conclusion:**

This case highlights the importance of remaining cognisant of the protean manifestations of extrapulmonary tuberculosis, and illustrates the advantage of a clinically directed multi-modality imaging approach to diagnosis.

## Background

Over the past decade, there has been a significant rise in the prevalence of extrapulmonary tuberculosis in the developed world, owing to the emergence of multi-drug resistant strains of *Mycobacterium tuberculosis *and an increasing number of immune compromised individuals and immigrants from the developing world [1-3]. Despite increasing awareness and the availability of better imaging and other diagnostic tests, extrapulmonary tuberculosis remains a difficult diagnosis to make, due to its often non-specific and protean manifestations. It is not uncommon for this disease to mimic malignancy. For instance, skeletal tuberculosis can clinically simulate sarcoma, leading to the referral of patients suffering from this condition to oncologists and surgeons, delaying correct diagnosis and the institution of appropriate therapy. Therefore, the prompt recognition by these clinicians of distinguishing features is vital for correct diagnosis, to facilitate timely anti-tuberculous therapy. In particular, imaging is a key tool in helping to make the diagnosis of extrapulmonary tuberculosis, through the recognition of certain key radiological patterns. However, as there are no pathognomonic imaging findings, the diagnosis ultimately rests on histopathological and microbiological confirmation. Whilst in the past this necessitated open surgical biopsy, tissue samples can now be obtained with minimal invasion, under image-guidance.

To highlight these issues we present the case of a young male with pain and swelling of the right elbow, referred to the Bone and Soft Tissue Sarcoma Service at our institution with a presumptive diagnosis of malignancy. Further investigation revealed extensive retroperitoneal abdominal and pelvic lymphadenopathy. The case illustrates the importance of a clinically directed multi-modality imaging approach for the exclusion of malignancy and the accurate diagnosis of tuberculous infection, by identifying sites of pathology and obtaining adequate tissue samples for histological and microbiological confirmation.

## Case presentation

### Case report

A previously well 21 year old East Timorese male presented to his local medical officer with a 2 month history of right elbow pain, associated with swelling and progressive limitation of movement. He also reported a 6 kg loss of weight during this time, but denied any other constitutional symptoms.

He appeared well, was afebrile, and had no abnormality on chest or abdominal examination. Examination of the right elbow revealed a firm, tender swelling, approximately 2 cm in diameter, over the lateral humeral epicondyle, with associated limitation of elbow flexion to 60 degrees.

**Figure 1:**
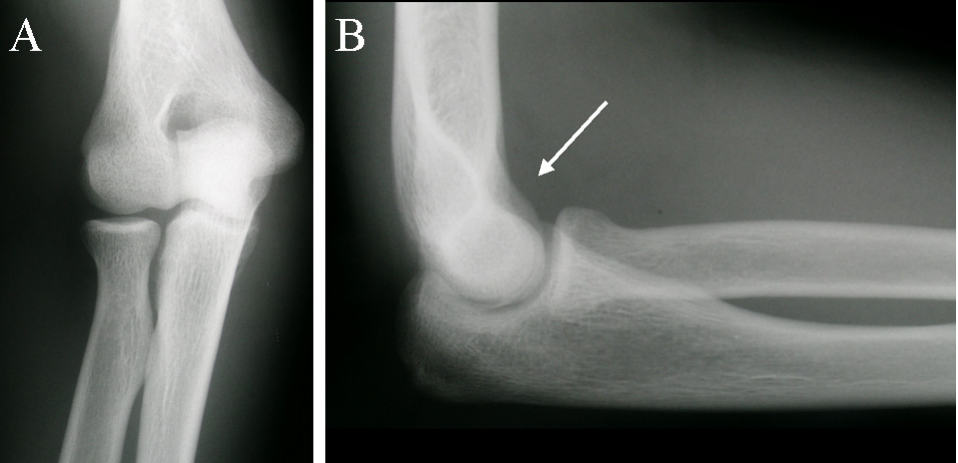
**Plain radiographs of the right elbow. **A. AP view, demonstrating no changes. B. Lateral view demonstrating a joint effusion (arrowed).

Initial investigation with plain XR of the right elbow joint revealed a joint effusion (Figure 1). As these findings were non-specific, further evaluation with different imaging modalities was undertaken.

**Figure 2:**
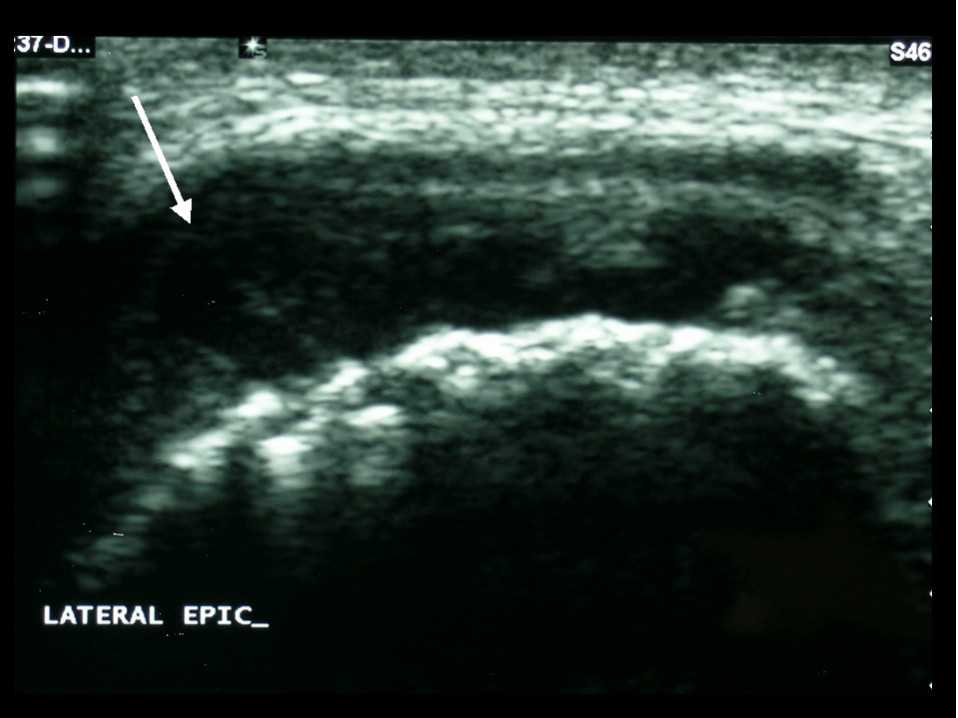
**Ultrasound examination of the right elbow. **This confirms the presence of a hypoechoic joint effusion (arrowed).

**Figure 3:**
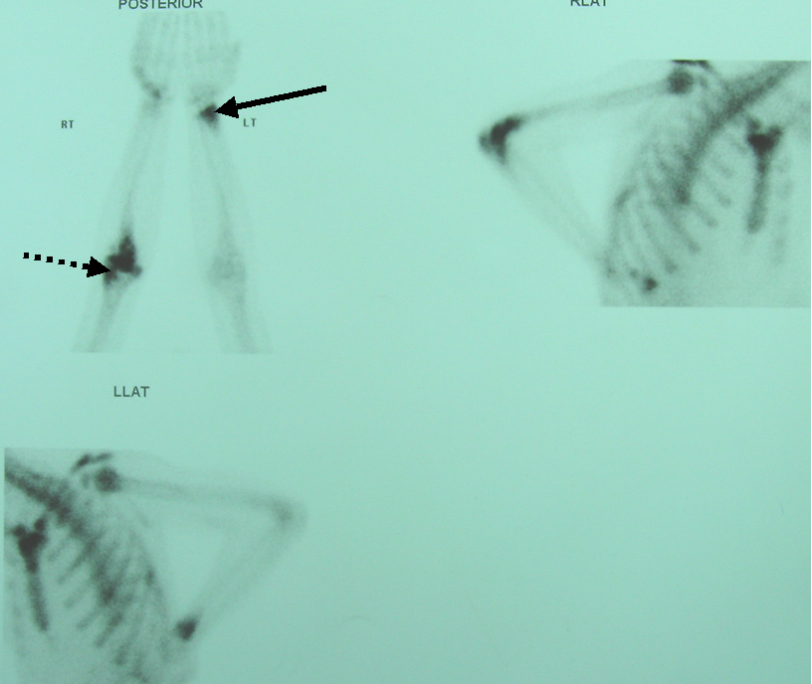
**Limited bone scan using Tc99m. **This demonstrates diffusely increased radioactive tracer uptake by the right elbow, particularly at the lateral epicondyle (dashed arrow) and the proximal ulna, as well as by the distal left radius (solid arrow).

Ultrasound examination of the elbow showed a large joint effusion (Figure 2). Scintigraphic imaging with Tc99m three phase bone scanning demonstrated increased blood pool activity, and diffusely increased osteoblastic activity, involving the right elbow joint, particularly the distal humerus and ulna, as well as the distal left radius (Figure 3).

**Figure 4:**
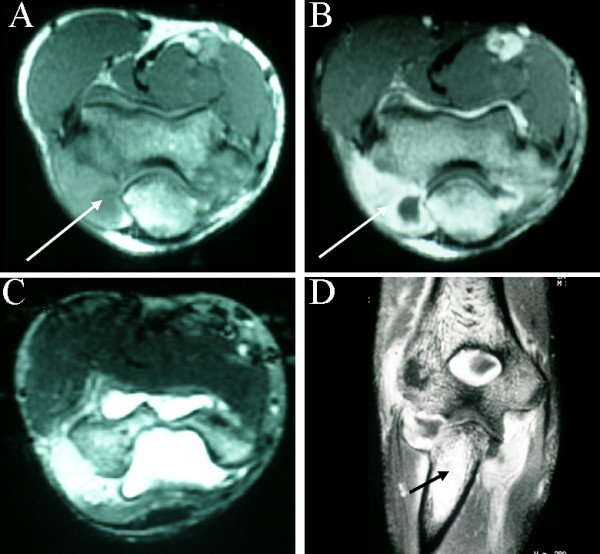
**Multiplanar CT of the right elbow joint. **A. Axial view showing a complex joint effusion (arrowed) and soft tissue swelling adjacent to the elbow. B. Axial slice through the proximal forearm, demonstrating the joint effusion surrounding the radial head (solid arrow) and increased soft tissue density involving the medullary shaft of the ulna (dashed arrow). C. Sagittal bone targeted reconstruction, showing subtle cortical erosions (arrowed) of the proximal ulna. D. Sagittal soft tissue reconstruction, demonstrating increased soft tissue density (arrowed) of the medullary shaft of the proximal ulna.

CT scan confirmed the presence of cortical erosions involving the right lateral epicondyle of the humerus and the ulna. Further, there was increased attenuation within the proximal medullary shaft of the ulna, and diffuse soft tissue thickening around the proximal ulna and radial head, with an associated joint effusion (Figure 4).

**Figure 5:**
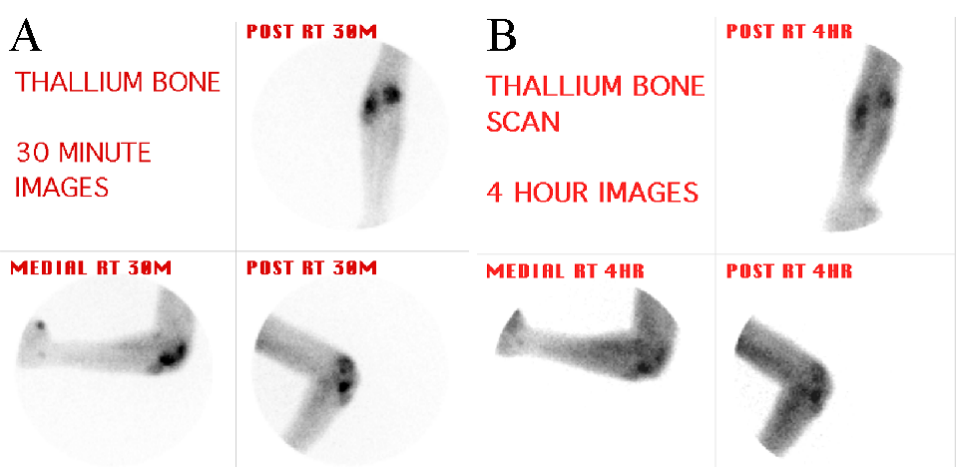
**Functional metabolic imaging, using Thallium 201 chloride. **A. Early planar images of the right elbow, showing intense radioactive tracer accumulation in the lateral humeral epicondyle and the proximal ulna. B. Delayed images at 4 hours, showing retention of tracer.

**Figure 6:**
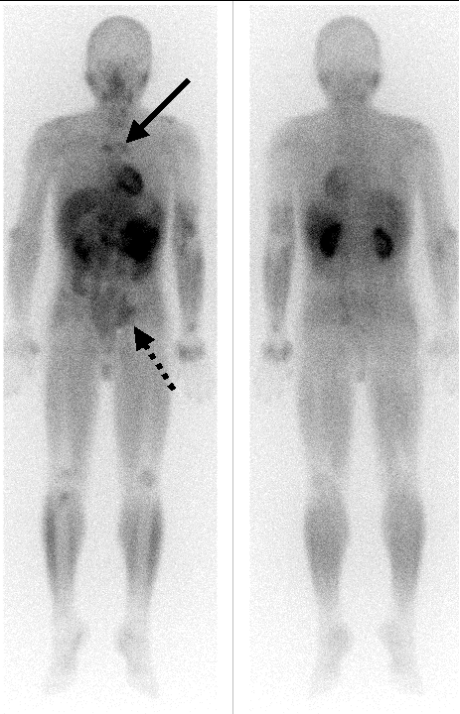
**Functional metabolic imaging, using Thallium 201 chloride. **Delayed whole body images, demonstrating abnormal tracer retention involving the mediastinum. (solid arrow), para-aortic region and left inguinal area (dashed arrow).

Further functional imaging with Thallium 201 chloride revealed intense early uptake in the right lateral epicondyle of the humerus and proximal ulna (Figure 5A). The delayed 4 hour images demonstrated tracer retention (Figure 5B). The delayed whole body views showed further abnormal thallium retention involving the mediastinum, para-aortic regions of the abdomen, the left inguinal region and the distal left radius (Figure 6).

**Figure 7:**
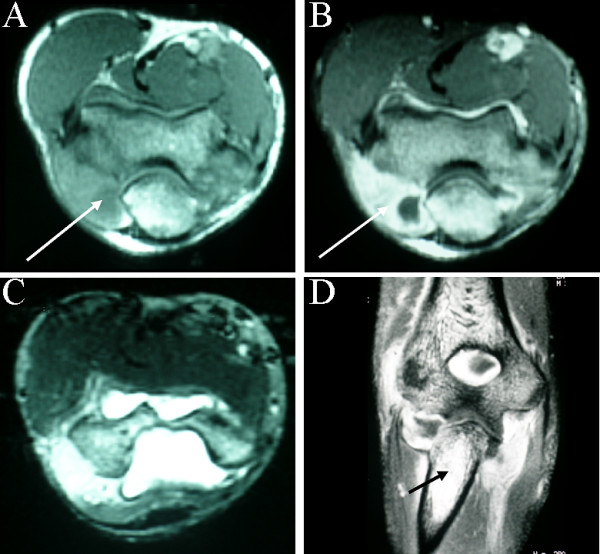
**Multiplanar MR imaging of the right elbow. **A. T1 weighted axial image at the level of the elbow joint, demonstrating a complex effusion (arrow), particularly adjacent to the proximal ulna. B. Contrast enhanced axial image showing enhancement around the joint (arrow). C. T2 weighted axial image again showing extensive joint effusion D. Coronal contrast enhanced image showing the complex effusion and abnormal signal intensity in the medullary canal of the proximal ulna (arrow).

MR demonstrated erosive changes involving the lateral epicondyle, a loculated joint effusion and thickening and enhancement of the joint capsule and the annular ligament of the superior radio-ulnar joint. These changes suggested either a neoplasm or possibly infection (Figure 7).

The patient was therefore referred to the Bone and Soft Tissue Sarcoma service for further investigation.

**Figure 8:**
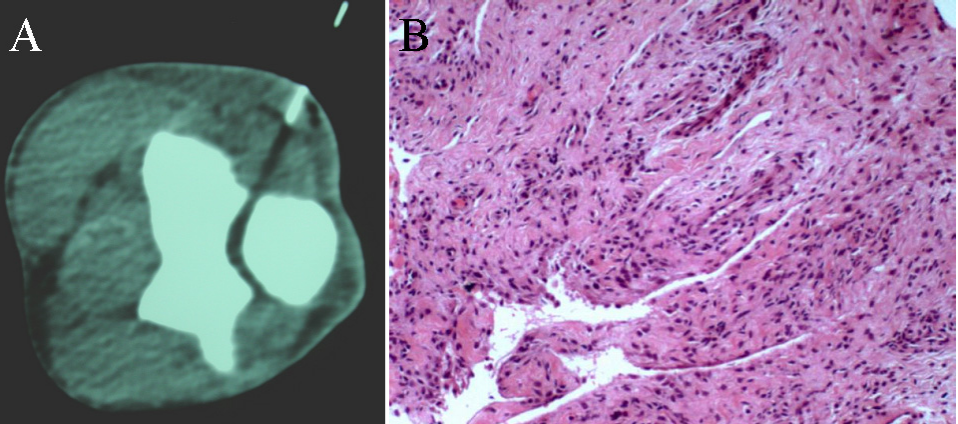
**CT guided core biopsy of the elbow. **A. Tissue adjacent to proximal ulna, at the level of the elbow joint, was obtained under CT guidance. B. Histological examination, after haematoxylin and eosin staining, demonstrated non-specific chronic inflammation and numerous polymorphonuclear cells.

Biopsy of the right elbow joint demonstrated chronic synovial inflammation and numerous polymorphonuclear cells in the synovial fluid, but no malignant cells (Figure 8). Microbiological examination, including staining for acid-fast bacilli and fungi, revealed no organisms.

**Figure 9:**
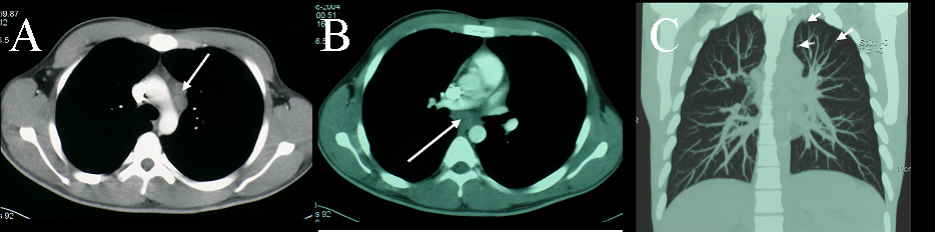
**Multiplanar chest CT. **This demonstrates mediastinal lymphadenopathy and subtle pulmonary involvement. A. Contrast enhanced axial slice, showing enlarged lymph nodes (arrowed) in the para-aortic window. B. Axial slice showing subcarinal lymphadenopathy (arrowed). C. Coronal slice, showing small nodules in the left upper lobe (arrowed).

**Figure 10:**
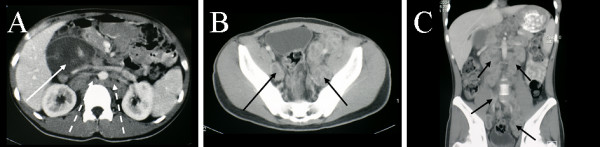
**Multiplanar abdominal and pelvic CT, demonstrating extensive lymphadenopathy. **A. Large low attenuation portal lymph node (solid arrow) showing peripheral enhancement, and associated para-aortic nodes (dashed arrows). B. Extensive heterogenous attenuation side wall pelvic adenopathy. C. Coronal image, demonstrating extensive low attenuation para-aortic and pelvic lymphadenopathy (arrowed)

CT scanning of the chest, abdomen and pelvis, performed in light of the thallium findings, revealed extensive retroperitoneal lymphadenopathy. The lymphadenopathy was characterised by peripheral enhancement with prominent central areas of low attenuation (Figures 9 and 10).

**Figure 11:**
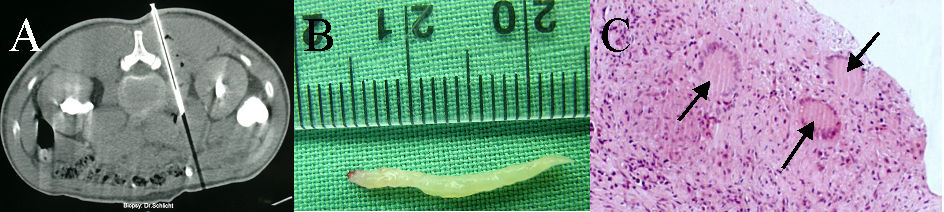
**CT guided biopsy of the para-aortic lymph nodes. **A. 14 gauge core needle biopsy. B. The core of tissue obtained. C. Histological examination after haematoxylin and eosin staining, demonstrating classic caseating granulomata of tuberculous infection (arrowed).

Subsequent CT guided biopsy of the retroperitoneal lymphoid tissue revealed necrotising granulomatous lymphadenitis indicative of Mycobacterium tuberculosis infection (Figure 11). While no acid fast bacilli were visible on Ziehl-Nielsen staining, polymerase chain reaction was positive for M. tuberculosis DNA. The lymph node biopsy specimens cultured M. tuberculosis after 6 weeks.

A final diagnosis of disseminated extrapulmonary tuberculosis was therefore made. Anti tuberculous therapy was commenced with good clinical response.

## Discussion

Extrapulmonary tuberculosis, a condition whose resurgence can be ascribed to the emergence of multi-drug resistance and an increasing number of immune compromised individuals and immigrants from the developing world, remains a difficult diagnosis to make [1-3]. It is one of the great mimickers in medicine, with non-specific clinical and radiological manifestations that can suggest numerous other disease entities in particular malignancy.

Although a positive tuberculin test and chest imaging findings are supportive of the diagnosis, absence of these does not exclude it [4,5]. Therefore other diagnostic tests need to be used, combined with a high index of clinical suspicion. As highlighted by the above case, a multimodality imaging approach assists in the early diagnosis of extrapulmonary tuberculosis, enabling the timely initiation of appropriate therapy.

Musculoskeletal involvement occurs in 1 to 3 percent of patients with tuberculosis, usually due to haematogenous seeding [4,6]. In more than 50 % of cases there is no evidence of concurrent active intrathoracic tuberculosis [4].

While the most common site of osseous involvement is the spine, followed by the femur, tibia and the small bones of the hands and feet, any bone can potentially be affected [1,4,6,9,10]. The most common presenting symptoms of tuberculous osteomyelitis are non-specific pain and swelling. Consequently, as in the above case, skeletal tuberculosis frequently mimics osteosarcoma, leading to incorrect initial diagnosis and delay in the institution of treatment.

Plain XR findings in bony tuberculosis include osteopenia, osteolytic foci with poorly defined edges, and varying amounts of sclerosis and periostitis [1,5,9]. The metaphysis is typically involved, although epiphyseal involvement also occurs [1]. A particular type of tuberculous osteomyelitis, cystic tuberculosis, produces round or oval radiolucencies with variable amounts of sclerosis [1,6]. These findings are, however, non-specific, and can be found in a range of pathological processes, including neoplasia. For instance, osteolytic and osteosclerotic foci can be found in metastases from various primary tumours, for instance prostate, breast and renal cell carcinoma, whilst sclerosis and a periosteal reaction may suggest a primary bone tumour. Even the radiographic characteristics of cystic tuberculosis can also be found in metastatic carcinoma or germ cell tumours, and plasma cell myeloma [1,4].

There are a few radiographic features, however, that favour tuberculous infection over neoplasia. These include the presence of small juxtacortical abscesses or rings of inflammatory tissue, due to cortical destruction and spread of infection to the extraosseous tissues [11]. Regardless, the features of tuberculous osteomyelitis are so variable and inconstant that further investigation is usually required.

Nuclear Medicine techniques of value in the evaluation of tuberculous bony involvement include Tc99m methylene diphosphonate bone scanning, particularly in multifocal bone involvement. Sensitivity is reduced significantly however in pure septic arthritis and discitis without concomitant osteomyelitis. In these clinical situations, MRI is the modality of choice.

Another useful radiotracer technique is functional metabolic imaging, which assesses the basal metabolic activity of inflammatory and neoplastic lesions. Thallium-201 chloride (TI-201) scanning is a good example of this modality. TI-201 is a potassium analogue that is actively concentrated in cells by the sodium-potassium ATPase pump and by a co-transport system mediated by a calcium-dependent ion channel [12]. As enhanced metabolic activity often increases the activity of these pumps, tumours frequently concentrate this tracer more avidly than normal soft tissue or bone, particularly on delayed images. Conversely, inflammatory processes usually show an early increase in tracer uptake, but with reduced metabolic activity, or a washout pattern, on the delayed images. Interestingly, tuberculous lesions often show significant delayed activity or retention as in this case.

Joint involvement in skeletal tuberculosis occurs commonly due to direct invasion from an adjacent focus of osteomyelitis, as in the case presented in this report [4,6]. Characteristically a monoarticular process, tuberculous arthritis usually involves the large weight bearing joints, namely the knee and the hip [1,5]. Phemister's triad of juxta-articular osteoporosis, marginal joint erosions and joint space narrowing is classically described as characteristic of this condition, however these features are again non-specific [1,13,14]. The multiplanar abilities of multislice of CT and MR are important in differentiating tuberculous arthritis from other disease processes. Bone targeted CT gives excellent bony detail, periosteal and cortical definition, while MR is the modality of choice for assessment of the presence and extent of bone marrow changes, effusions and synovial involvement.

Abdominal tuberculosis, like musculoskeletal involvement, is a difficult diagnosis to make, due to its varied clinical presentations, and is again often mistaken for malignancy. Lymphadenopathy is the most common manifestation, with up to two thirds of patients with abdominal tuberculosis demonstrating lymph node involvement [15,16]. The mesenteric, omental and peripancreatic groups most frequently involved, reflecting the lymphatic drainage of the most commonly affected sites in the small bowel and liver [1,16]. Lymph node involvement is usually detected on CT imaging, and this is also the imaging modality of choice for the evaluation of intraabdominal and pelvic lymphadenopathy [16,17]. The main differential diagnosis for diffuse lymph node enlargement is lymphoma, whilst less widespread intraabdominal lymphadenopathy may suggest metastatic malignancy [16].

Certain features on CT imaging help distinguish tuberculous lymphadenopathy from these neoplastic causes. In tuberculous infection, the nodes are usually multiple and large, averaging 2 to 3 cm in diameter [1]. Peripheral enhancement with central areas of low attenuation or loculation are seen on contrast enhanced CT in the majority of cases [6,16-18]. Conglomerate mixed density nodal masses may also occur, likely representing multiple confluent nodes with peri-nodal spread of inflammation [16]. In contrast, lymphomatous adenopathy is characteristically associated with homogenous attenuation [19]. However, whilst heterogeneity is characteristic of the caseous necrosis seen in tuberculous lymphadenopathy, it is by no means pathognomonic, also occurring in metastatic testicular carcinoma [16]. Likewise, although nodal calcification is highly suggestive of tuberculous disease, especially in endemic areas, it can also occur in teratomatous testicular metastases and in non-Hodgkin's lymphoma after treatment with radiotherapy [16].

Because of this overlap in imaging appearances between extrapulmonary tuberculosis and malignancy, even in cases where the imaging and clinical features strongly suggest tuberculosis, the diagnosis requires histopathological and bacteriological confirmation. In the past, this often necessitated open surgical biopsy. Now core needle biopsy has been shown to be quick, safe and effective alternative for obtaining tissue specimens under image guidance [20].

## Conclusion

The clinical and radiologic features of extrapulmonary tuberculosis often mimic those of many other pathological processes, in particular malignancy. Oncologists and surgeons must therefore remain cognisant of the possibility of tuberculous infection, and of radiological features that help distinguish this condition from neoplastic processes. However, even with the use of multiple imaging modalities and laboratory diagnostic tools, a definitive diagnosis still requires a positive culture or histologic analysis of biopsy specimens in many cases. Image-guidance facilitates optimal tissue samples to be obtained for analysis.

## Abbreviations

Tc99: Technicium 99

TI-201: Thallium 201

TB: tuberculosis

## Competing interests

The author(s) declare that they have no competing interests.

## Authors' contributions

SAA drafted and revised the manuscript, and was involved in the editing of the images.

PFC, PJS, GJP and JS critically evaluated and revised the manuscript. They also provided information from their respective areas of expertise, which was critical to the discussion section of the manuscript.

SMS was responsible for the conception of this case report, and was also involved in the drafting and revision of the manuscript.

All authors have read and given final approval of the submitted version of the manuscript.
